# Development of a FRET-based fluorescence aptasensor for the detection of aflatoxin B1 in contaminated food grain samples

**DOI:** 10.1039/c8ra00317c

**Published:** 2018-03-14

**Authors:** Aswani Kumar Y. V. V., Renuka R. M., Achuth J., Venkataramana Mudili, Sudhakar Poda

**Affiliations:** Department of Biotechnology, Acharya Nagarjuna University Nagarjunanagar Guntur Andhra Pradesh-522510 India sudhakarpoda@gmail.com; DRDO-BU-CLS, Bharathiar University Campus Coimbatore Tamil Nadu-641046 India ramana.micro@gmal.com +91-422-2428162

## Abstract

The present study aimed to develop an aptamer-based FRET detection strategy for the specific and sensitive detection of AFB1 in contaminated food grains. The study comprises generation of ssDNA aptamers against AFB1 by whole-cell SELEX and their application in a FRET-based platform utilizing graphene oxide (GO) and quantum dots (QDs). The generated aptamers were characterized to determine their specificity and sensitivity using indirect ELISA where AFB1–OVA was used as a coating antigen. Among the aptamers generated, the ATB1 aptamer showed good reactivity and selectivity against AFB1. This aptamer was further characterized to determine its secondary structure and KD value, which was found to be 5.9 kcal mol^−1^. The characterized aptamers were conjugated onto Cd/Se quantum dots to develop a fluorimetric system for the detection of aflatoxin B1 using a graphene oxide platform. The presence of graphene oxide quenches the fluorescence ability of the quantum dots due to π–π stacking interactions between the aptamer and GO. Upon target addition, the aptamer forms a complex with aflatoxin B1 thereby restoring the fluorescence intensity. The developed assay shows a linear response from 0.002 μg μl^−1^ to 0.2 μg μl^−1^ with a detection limit of 0.004 μg μl^−1^ for the AFB1 standard toxin and showed no cross-reactivity with other closely related mycotoxins. To validate the reliability of the developed method, several field samples spiked with AFB1 were included in this study and the results obtained were cross verified using a standard commercial AFB1 kit. In conclusion, the developed method may find good utility in routine food testing laboratories for risk assessment of AFB1.

## Abstract 2

The present study aimed to develop an aptamer-based FRET detection strategy for the specific and sensitive detection of AFB1 in contaminated food grains.

## Graphical abstract

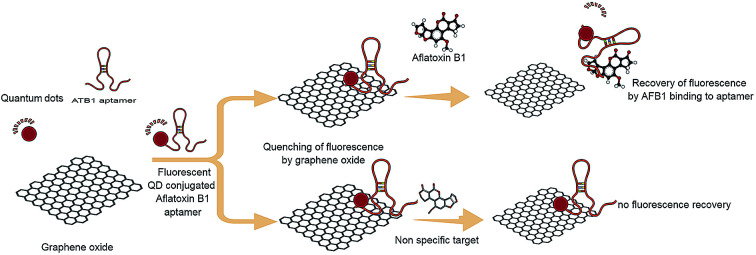

## 1. Introduction

Food safety has grown to be an important global issue with implications for public health and international trade since foodborne outbreaks from microbial contaminants, chemicals, and toxins have provoked major health crises. Contamination of agricultural products by fungi that can produce toxic metabolites known as mycotoxins is of high importance due to its impact on the health of humans and livestock. Regardless of the control measures followed, mycotoxins are present in human and animal feeds and are reported to infest before harvest, post-harvest or during processing and storage, affecting the quality grade of foods.^1,2^ Among the reported mycotoxins, aflatoxins are the most widespread group of toxins that cause food product contamination, and are secondary metabolites produced by *Aspergillus sps* under certain conditions. The four main aflatoxins include aflatoxin B1, B2, G1, and G2 produced by *Aspergillus parasiticus* and *Aspergillus flavus*. Aflatoxin B1 is known to be highly toxic and responsible for human hepatocellular carcinoma, and is listed as a group I carcinogen by the International Agency for Research in Cancer.^3^ The European Union has established the maximum allowed level of aflatoxin B1 in foodstuffs like milk, dried fruits, and groundnuts as 2 ng ml^−1^.^4^ Prevention of aflatoxin B1 contamination is of economic importance due to its pivotal role in health concerns for human and livestock as well as the marketability of agro-products. With the gradual increase in the incidence of aflatoxin B1 food contamination, researchers have been incited to develop a simple and economical method for the detection of aflatoxin B1 in food and environmental samples.^5^

The most widely employed techniques for analysis of mycotoxins include high-performance liquid chromatography (HPLC), gas chromatography/mass spectrometry (GC/MS) and probe-based immunochromatographic assay. Conventional ELISA requires time-consuming incubation and washing protocols whereas enzyme-based labels suffer from denaturation and degradation issues. The techniques developed to date involve complex instrumentation and multifaceted protocols for sample preparation, and are quite time-consuming.^6^

Among the abundance of nanomaterials, two-dimensional nanomaterials have emerged as promising platforms with enhanced light absorbance and electron transfer rates. Carbon sources are of great interest and are employed in the fabrication of various sensing platforms. Compared to graphite and glassy carbon, graphene oxide exhibits the characteristic of chemical inertness, which makes it a material of choice for biosensor development.^7^ Other biosensor materials include graphene derivatives, chalcogenides, and metal oxides. Graphene, a sheet made of sp^2^ bonded carbon atoms arranged into a rigid honeycomb lattice, has increased mechanical strength compared to the materials reported so far. Additionally, graphene provides enhanced electron transfer capabilities and conductivity, unique pliability, and constructive biocompatibility.^8,9^ All these properties endorse the application of graphene in sensing devices. Designing aptasensors with graphene will satisfy the necessary requirements for achieving high-performance aptasensors. Graphene exhibits noncovalent interactions with aptamers through π–π stacking interactions between the purine and pyrimidine bases.^10–12^ Based on this, many inventive strategies have been developed for aptasensor platforms.^13^

Aptamers are emerging synthetic materials selected through the systematic evolution of ligands by exponential enrichment (SELEX) technique, which has drawn wide appreciation from both the theoretical and experimental scientific communities since its discovery in 1990.^14,15^ Aptamers can bind to a diverse range of targets from small organic molecules to biological macromolecules. As an alternative to antibody-based systems, aptamers offer several advantages, including thermal and chemical stability, practical synthesis, increased binding affinity, and flexible modification with functional groups like thiols and amines. These exceptional properties make aptamers the material of choice for highly sensitive biosensing platforms (aptasensors). Ever since the discovery of aptamers for use against mycotoxins in 2008, a variety of aptasensing platforms utilizing electrochemiluminescence, fluorescence and colourimetric transducers have been reported for the detection of mycotoxins.^16^ Integrating the structural properties of nanomaterials with the highly specific recognition abilities of biomolecules has created a unique way to analyse target analytes in food and environmental samples.

Forster or Fluorescence Resonance Energy Transfer (FRET) has been used to investigate various molecular interactions and gather information about nanoscale processes for the past few decades and remains an important technique employed in the field of biomedical analysis. FRET involves energy transfer from donor fluorophores to acceptor fluorophores by means of intermolecular dipole–dipole coupling.^17^ It is a highly sensitive technique, where energy transfer will only occur if the donor and acceptor remain within a distance limit of 10 nm. In FRET assays, separate donors and acceptors are brought together into close proximity through antibody/aptamer–antigen interactions. These assays generally utilize organic dyes due to their simpler preparation protocols. However, the typical organic dyes employed in the assays suffer from the limitations of low chemical stability and photo-bleaching, which deteriorate the reliability of the sensors.^18^ In recent years, quantum dots have been employed in detection platforms owing to their size-tunable spectra, stability, and high quantum yields. Moreover, conjugation of QDs to aptamers can be achieved without affecting the emission properties or the specificity of the aptamer.^19,20^ Developing novel fluorescence-based techniques for probing biomolecules in combination with aptamers could give rise to more opportunities for aptasensors with enhanced performance.

Considering the advantages of the FRET-based assay, we developed a platform utilizing the properties of FRET between quantum dots and graphene oxide. The interactions between ssDNA, GO and the target were combined together to develop a sensitive and selective method for a fluorescence quenching based detection platform. Biotin-modified ssDNA aptamers specific towards aflatoxin B1 were first conjugated to orange Cd/Te QDs. The interactions between the aptamers and GO in the absence of the target analyte quench the fluorescence of the attached QDs. Upon recognition of the target, the increasing distance between the QDs and GO and the destabilized aptamer–GO interactions enhance the fluorescence of the QDs. Detection of AFB1 in the system will elicit aptamer–AFB1 complex formation, resulting in the release of fluorescence from the QDs. The fluorescence varies according to the concentration of aflatoxin B1 in solution. The developed method was validated using spiked food grains and the results obtained were cross verified using a commercially available aflatoxin ELISA kit (MyBioSource, US).

## 2. Materials and methods

### 2.1 Chemicals, media and reagents

All chemicals used were of analytical grade or of the highest purity available. Salts were purchased from Himedia Laboratories (India), except those mentioned specifically. The aptamer library (1 μM), primers and biotinylated probes (both 25 nM) were synthesized at IDT (US). The stock and working dilutions of the library and the primers were maintained in MilliQ water. PCR buffers, Taq DNA polymerase and T4 DNA ligation enzymes were purchased from NEB (USA), and the pGEM-T vector was purchased from Promega (US). Graphite powder, cadmium oxide and tellurium were purchased from Sigma-Aldrich (USA). The nitrocellulose membrane (0.45 μm) (GE Healthcare, Germany), aflatoxin B1, thioglycolic acid (TGA, Sigma-Aldrich), *N*-hydroxysuccinimide (NHS), 1-(3-dimethylaminopropyl)-3-ethylcarbodiimide hydrochloride (EDC, Sigma-Aldrich), and streptavidin (Himedia) were used without any further purification. A 0.1 M phosphate buffer solution (PBS) of pH 7.4 and a carbonate bi-carbonate buffer of pH 8 were used in the following experiments.

### 2.2 DNA library and primers

The ssDNA library (1000 nmol), with 45 nucleotides comprising central random regions flanked by constant regions at the 3′ and 5′ ends, was purchased from IDT technologies (US). Forward and reverse primers (Table 1) were used to amplify the pool and a biotinylated reverse primer was employed to label the aptamers.

**Table 1:** Primers used in the generation of aptamers, and the selected aptamer sequences (ATB1–ATB4 aptamer)

Name	Sequence (5′–3′)	Synthesis Scale
SelexAPLIB2	ATAGGAGTCACGACGACCAGAANNNNNNNNNNNNNNNNNNNNNNNNNNNNNNNNNNNNNNNNTATGTGCGTCT ACCTCTTGACTAAT	1 μM
Apta F1 N	ATAGGAGTCACGACGACCAGAA	
Apta R1 N	ATTAGTCAAGAGGTAGACGCACATA	250 nM
Apt Bio Rev	5Biosg/ATTAGTCAAGAGGTAGACGCACATA	250 nM
		
**Selected aptamer sequences after different rounds of SELEX**		
ATB1	ATATCTTTTCCTACTCATCTTTGAATAACTACCGGGCATTACTTTCTGGCCTCCCTGCCTCCTAAATCACCAATTAATTCGCGGCCCCCCG	
ATB2	CGTGATAATTGGCGCCGACTTCGCATGCTCCCCGCCGCCATGGCGGCCGTGAGAATTAGATTATAAGAGTCCCCACGACCAT	
ATB3	ATTTGGGGCCGACGTCGCATGCTCCCGGCCGCCATGGCGGCCGCGGGAATTCGATTATTGGAATCGAGGCCACCATAAATA	
ATB4	TATACGTTCCTCTGAACATGATCACCTCTCTGGTTCTCTCCGATCTGGTCCACCTGGTGTGAGAGGCGATTCTGCATTAGCTA	

### 2.3 Generation and characterization of AFB1 aptamers

#### 2.3.1 Generation of anti-AFB1 aptamers

A binding buffer (50 mM Tris-Cl (pH 7.2), 5 mM KCl, and 150 mM NaCl) was used throughout the SELEX process for equilibration and washes. The AFB1 specific aptamers were generated through the SELEX process by utilizing immunoaffinity columns. Columns packed with anti-AFB1 antibodies were incubated with 75 ng μl^−1^ crude toxin for 30 min and this step was repeated 4–5 times to achieve a high binding efficiency. To these toxin packed columns, single-stranded DNA oligos of nearly 150 pmol were added and incubated for an hour followed by denaturation at 95 °C for 10 min. Unbound fractions were removed through repeated washing with buffer. Finally, the bound oligonucleotides were eluted with prewarmed 1× PCR buffer. The eluted fraction was used as a template and a PCR reaction was carried out (denaturation for 15 s at 95 °C, then annealing at a temperature of 57 °C for 30 s and extension at 72 °C for 45 s with 30 cycles each). The cycle was repeated nearly 10 times with alternate cycles of SELEX and counter SELEX from round 6. Counter SELEX was performed to enhance the specificity of the pool against the target. The final pool was cloned in a pTZ57R/T cloning vector using Express Link T4 DNA ligase (Invitrogen, USA). Ligated samples were transformed into *E. coli* DH5α cells. The obtained clones were tested using colony PCR using T7 forward and reverse primers. Positive clones with increased specificity towards AFB1 were sequenced and secondary structures were predicted using M-fold software.^21^

#### 2.3.2 Characterization of generated aptamers

The selected aptamers (ATB1, ATB2, ATB3 and ATB4), which are specific towards aflatoxin B1, were generated using a column based on the SELEX procedure. The sensitivity and specificity of these aptamers were determined by indirect ELISA. Briefly, the AFB1–OVA toxin (1 μg per well) in carbonate–bicarbonate buffer (pH 9.6) was coated onto microtiter plates and incubated at 37 °C for 1 hour. 3% BSA (w/v) in 1× PBS was used to block the unoccupied sites. The aptamers were biotinylated and diluted from 1 : 500 to 1 : 64 000 in PBS and incubated at 37 °C for an hour. After washing with PBST and PBS three times, plates were incubated with a streptavidin–HRP conjugate (1 : 2000 dilution) at 37 °C for 1 hour. The plates were developed using TMB (Bio-Rad, USA) and read at 450 nm. The initial library pool and PBS were used as the controls. Similarly, the specificity of ATB 1, 2, 3 and 4 was determined by performing indirect ELISA on the microtiter plate for closely related toxins such as AFB2, ochratoxin A (OTA), citrinin, deoxynivalenol, fumonisin B1 and PBS (negative control). Among these, the highly reactive and specific aptamers were employed to develop the detection platform and were analyzed further to determine their dissociation constants using M-fold software.

### 2.4 Synthesis and characterization of CdTe quantum dots

#### 2.4.1 Synthesis of CdTe quantum dots

Highly fluorescent and water-soluble CdTe quantum dots were synthesized *via* the method described earlier with the aid of microwave irradiation. Borate–acetic acid buffer (17 mM glacial acetic acid, 17 mM sodium tetraborate decahydrate) was used to carry out the reaction under ambient atmospheric conditions. In a round-bottom flask 1 mM CdCl_2_, 3 mM methane sulfonic acid and 0.2 mM sodium tellurite, in a ratio of 5 : 15 : 1, were mixed in 100 ml of the above-mentioned borate buffer to prepare the precursor solution at room temperature. After vigorous stirring, sodium borohydride was added to the precursor solution and the pH was adjusted to 9.0 with stirring. High-quality CdTe quantum dots were prepared using microwave irradiation at 600 W. The product was precipitated with ethanol and centrifuged at 6000 rpm for 10 min. The sediment obtained was resuspended in borate buffer.^22–24^ TEM micrographs were recorded at 200 kV using a JEM 2010 electron microscope. Zeta potential and particle size distribution measurements were carried out using differential light scattering using a Malvern Zetasizer.

#### 2.4.2 Surface modification and bioconjugation of quantum dots with AFB1 aptamers

The synthesised QDs were centrifuged at 3000 rpm for 5 min and the resulting pellets were dissolved in chloroform. Surface modification was carried out in borate buffer at pH 9, with the addition of TGA, and the solution was vortexed for 30 min. An ethanol wash was carried out to remove excess TGA. The resulting surface modified QDs were precipitated with ethanol and resuspended in borate buffer for further application. The prepared water-soluble surface modified CdTe quantum dots were activated using simple carbodiimide chemistry to enable conjugation with the biotinylated aflatoxin B1 aptamers. Around 1 ml of QDs in PBS (0.01 mol l^−1^, pH 7.2) was stirred along with 100 μl of EDC and 100 μl of NHS, in a ratio of 1 : 4, for 1 hour to activate the free carboxylic acid groups on the surface of the QDs. Streptavidin (1 mg ml^−1^) was added to the activated QDs and stirred for an hour at ambient temperature.^25^ Biotinylated aflatoxin B1 aptamers were added into the system containing streptavidin functionalized QDs and incubated for an hour at room temperature with uniform stirring. Unbound aptamers were removed using centrifugation at 6000 rpm for 10 min. The pellets obtained were washed with ultrapure water and finally resuspended in borate buffer to be stored at 4 °C for further studies.

### 2.5 Preparation of GO

Graphene oxide (GO) was prepared *via* the reported Hummer’s method with slight modification. In brief, 5 g of graphite powder and 2.5 g of sodium nitrate were pre-oxidized through stirring with 50 ml of concentrated H_2_SO_4_ in an ice bath for 30 min. The resulting suspension was washed with deionized water and vacuum dried at 50 °C. Further oxidation was carried out *via* addition of pre-oxidized graphite to a mixture of concentrated H_2_SO_4_/H_3_PO_4_ and 9 g of KMnO_4_ with constant stirring. The reactants were allowed to cool to room temperature followed by the addition of 30% H_2_O_2_. The resultant product was filtered and the filtrate was subjected to centrifugation at 5000 rpm for 30 min. After subsequent washes with water and ethanol, the final sediment was vacuum dried and the GO obtained was stored as a solid brown powder. The prepared GO was characterized using SEM and TEM to determine its morphology, followed by FTIR spectroscopy and TGA to determine its structural properties.

### 2.6 Aptamer based quencher assay for aflatoxin B1 detection

Various concentrations of GO (0.01 mg ml^−1^ to 1 mg ml^−1^) were mixed with the AFB1 aptamer–QD conjugates in 1 ml of phosphate buffered solution (pH 7.2). BSA with a final concentration of 0.5% was added to the above mixture and incubated for a period of time to allow quenching of the quantum dot–aptamer conjugates. The optimal concentration of GO and quenching time were recorded. The absorption and emission spectra were recorded using a Biotek Synergy H1 Hybrid Multi-Mode Reader and Shimadzu RF 5301 spectrofluorometer with toluene and water as the solvents for the QDs and modified QDs, respectively.

### 2.7 Detection of aflatoxin B1 with the AFB1–QD–GO quencher system

The optimized assay was validated by analyzing the sensitivity of the developed platform with various concentrations of aflatoxin B1. Toxin concentrations ranging from 0.2 μg μl^−1^ to 0.002 μg μl^−1^ were added to the aptamer–QD–GO quenching system with gentle shaking at room temperature. Fluorescence spectra were recorded to evaluate the fluorescence recovery ability of the developed system in the presence of the target. Food samples contaminated with aflatoxin B1 were analyzed using this system and the results were compared with those from standard indirect ELISA.

### 2.8 Evaluation of the developed system with spiked samples

Maize and wheat samples were purchased from local markets and tested for the presence of aflatoxin B1. To perform spiking studies, the food samples were finely ground and spiked with a known concentration of AFB1 to achieve concentrations of 1, 2, 4, and 8 μg kg^−1^. The spiked samples were dried overnight and extracted with methanol and water in a ratio of 20 : 80. The resultant mixture was centrifuged and the supernatant obtained was filtered through 0.45-micron filters. The filtrate obtained was diluted fourfold with deionized water to reduce matrix effects and utilized directly for the FRET aptasensor assay.

### 2.9 Statistical analysis

All experiments were repeated three times with similar conditions to ensure the reliability of the data. Results were presented as mean values with standard deviation (SD). Statistical differences between treatments were analyzed using univariate (ANOVA) and the Student’s ‘*T*’ test to determine the level of significance of the data generated and were plotted using Graph Pad Prism6.

## 3. Results

According to an estimate proposed by the Food and Agriculture Organization, 25% of the world’s food crops are contaminated with mycotoxins every year. Being a toxic metabolite produced by fungi, mycotoxins contaminate globally consumed cereals like maize and wheat. Aflatoxins remain the most toxicologically relevant mycotoxins causing liver cirrhosis or primary liver carcinoma and are immunosuppressive. With varying environmental conditions, ideal systems to analyze these toxin outbreaks are highly essential.

### 3.1 Generation and characterization of AFB1 aptamers

#### 3.1.1 Generation of AFB1 specific aptamers

**Fig. 1:**
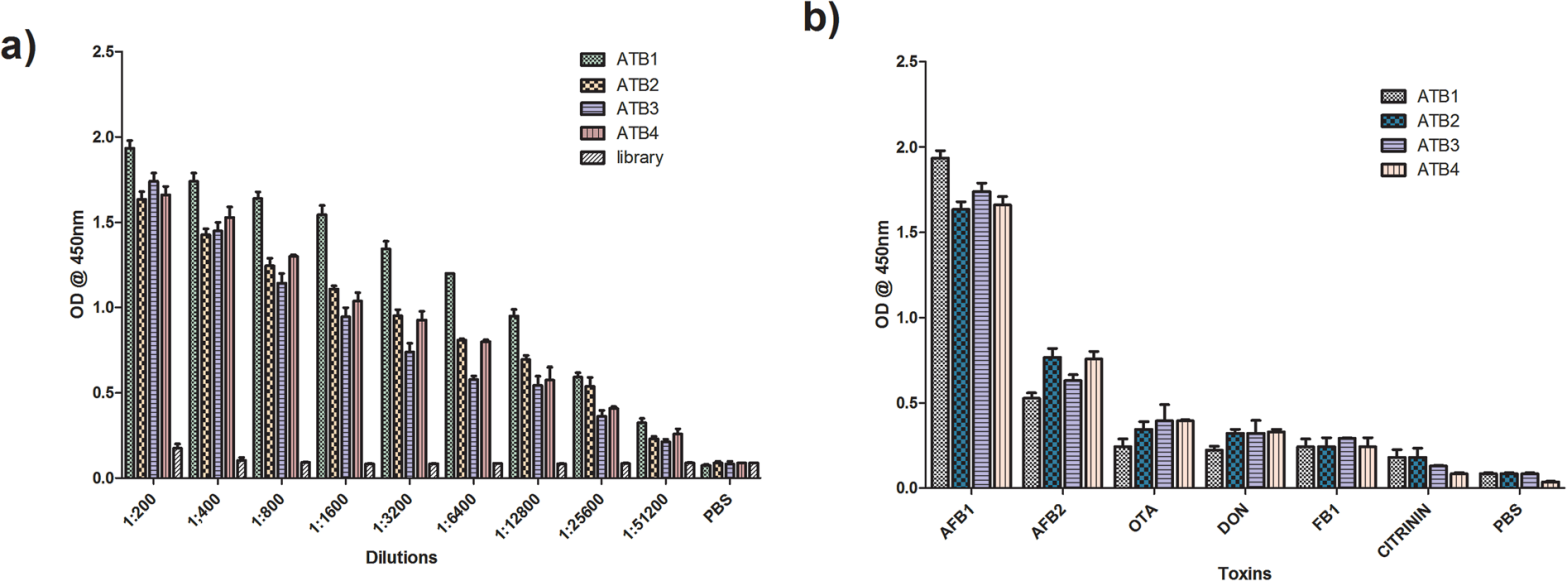
Characterization of the generated aptamers against AFB1. (a) Aptamer (ATB1–ATB4) titre values determined through indirect ELISA, and (b) specificity of the generated aptamer pools against closely associated mycotoxins.

**Fig. 2:**
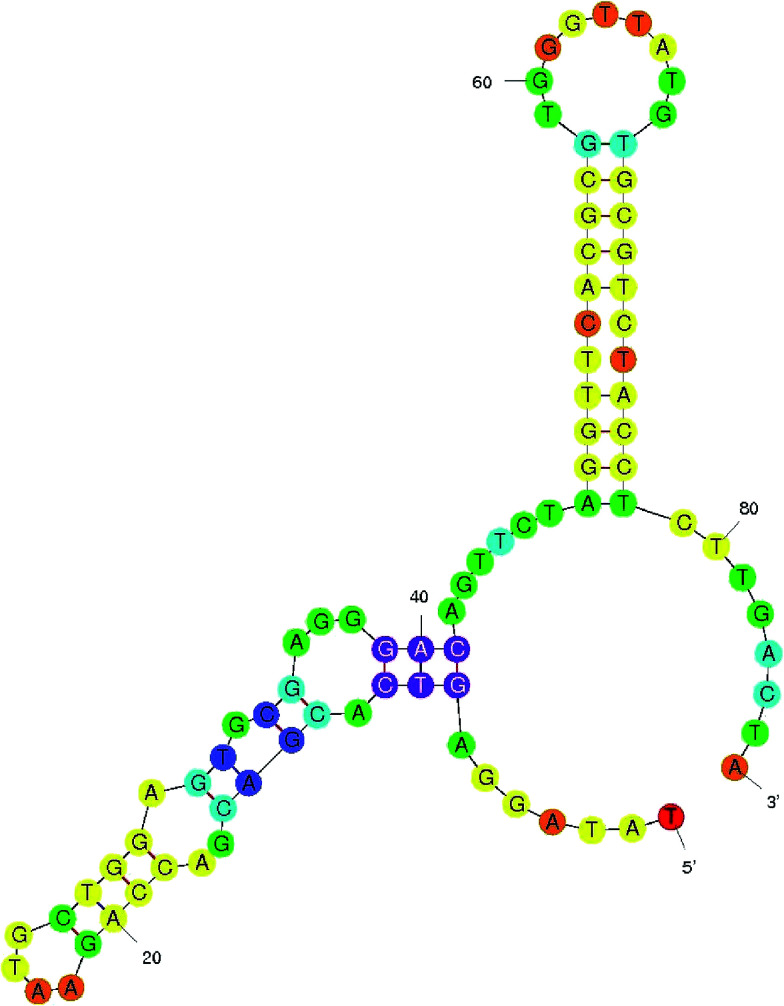
Aptamer secondary structure. The secondary structure and dissociation constant of the selected aptamer ATB1 were predicted using M-fold software.

Aptamers specific to AFB1 were generated through immunoaffinity column based SELEX with five rounds of SELEX and three rounds of counter SELEX. The un-reactive pools/non-specific pools accumulated and were eliminated through stringent washing steps as well as counter SELEX. Hence the final pool obtained consisted of highly specific aptamers, which were subjected to asymmetric PCR with an increased extension time (15 min), to achieve poly A tagging at both ends. The amplified aptamer pools were cloned in a pGMET cloning vector; transformed positive colonies were recovered and plasmids were purified and sequenced for target aptamer sequences (Table 1). The generated aptamers, ATB1, ATB2, ATB3 and ATB4, against aflatoxin B1 were characterized to determine their specificity and sensitivity. The titer value of the ATB1 aptamer was found to be the highest as determined through indirect ELISA against an AFB1–OVA conjugate (Fig. 1a). The obtained dilution corresponds to an ATB1 aptamer concentration of 150 ng ml^−1^, and the same was employed for further assays. ATB1 showed cross-reactivity against AFB2 but this was significantly lower than that of other aptamers, and moreover was significantly lower than that of the AFB1 toxin (Fig. 1b). An M-fold server was used to predict the secondary structures and perform the free energy calculations. The predicted secondary structure of the ATB1 aptamer is shown in Fig. 2, and the free energy of ATB1 was determined to be 5.9 kcal mol^−1^.

### 3.2 Generation and characterization of nanomaterials

#### 3.2.1 Synthesis of graphene oxide

**Fig. 3:**
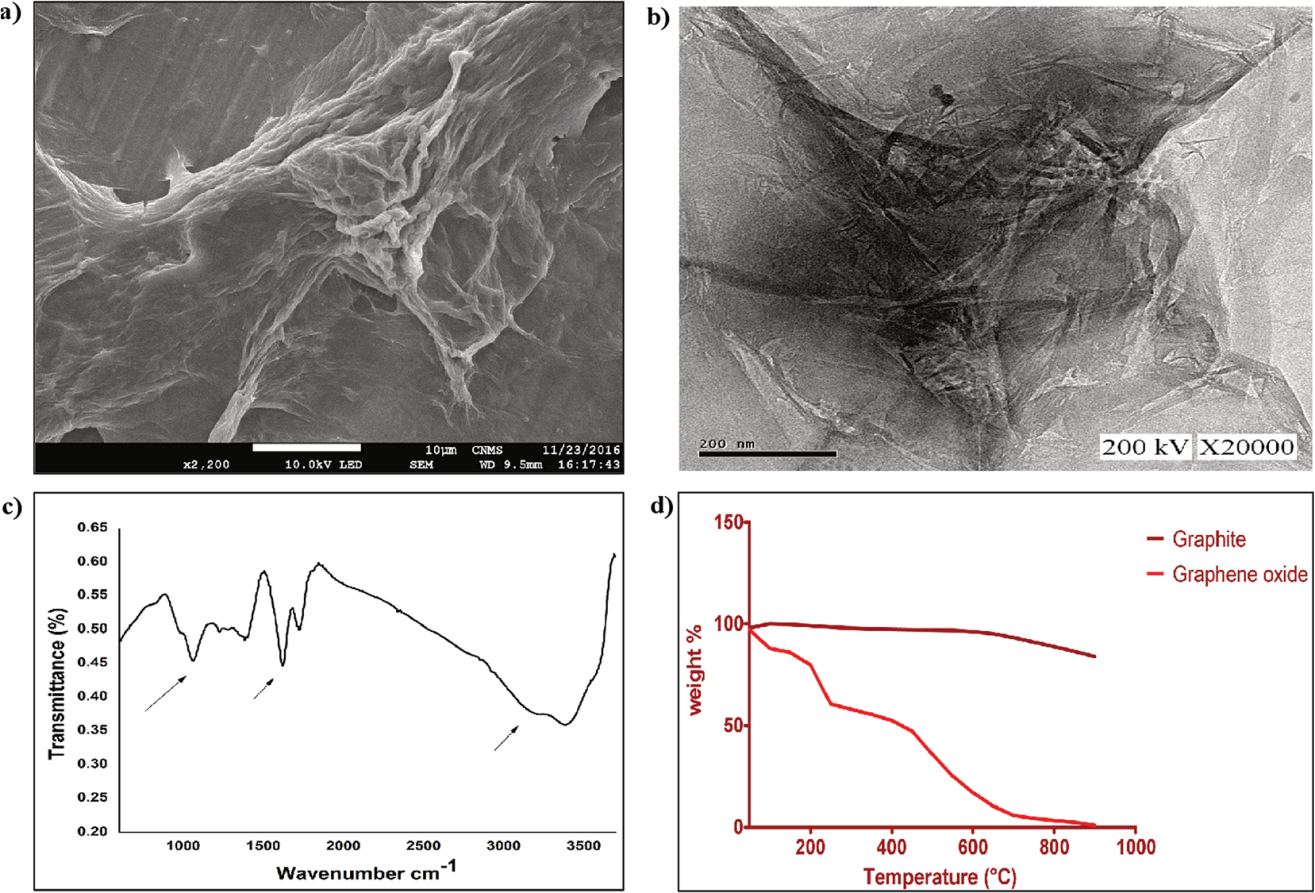
Characterization of graphene oxide. Surface characterization of synthesized graphene oxide using (a) SEM and (b) TEM, (c) FTIR analysis for functional group determination, and (d) TGA to determine the stability of the synthesized GO.

Soluble graphene oxide was prepared from graphite using a modified Hummer’s method. The synthesized GO exhibits a single layered morphology, possessing crinkles with rolled edges and a thickness of 2 nm that was confirmed using SEM and TEM analysis (Fig. 3a and b). Additionally, the FTIR spectrum in Fig. 3c shows characteristic peaks corresponding to the presence of different functional groups, such as hydroxyl, carboxylic acid and also ester, as reported in previous studies^31^ (broad peak of O–H at around 3300 cm^−1^, C=O stretch at 1730 cm^−1^, C=C stretch at 1616 cm^−1^, and alkoxy C–O stretch at 1051 cm^−1^ (Fig. 3c)). TGA was performed on graphite and graphene oxide from 50 °C to 900 °C at 10 °C min^−1^ under a nitrogen supply. From Fig. 3d, it was observed that graphene oxide is thermally unstable compared to graphite and started losing weight even below 100 °C (Fig. 3d). Two significant drops in GO mass were observed around 250 and 550 °C due to decomposition of oxygen-containing groups into CO and pyrolysis of the carbon backbone, respectively.^32^ The synthesised graphene oxide dispersed well in aqueous solution at a concentration of 1.5 mg ml^−1^, and was used further for FRET assays.

#### 3.2.2 Synthesis and characterization of CdTe quantum dots and their bioconjugates

**Fig. 4:**
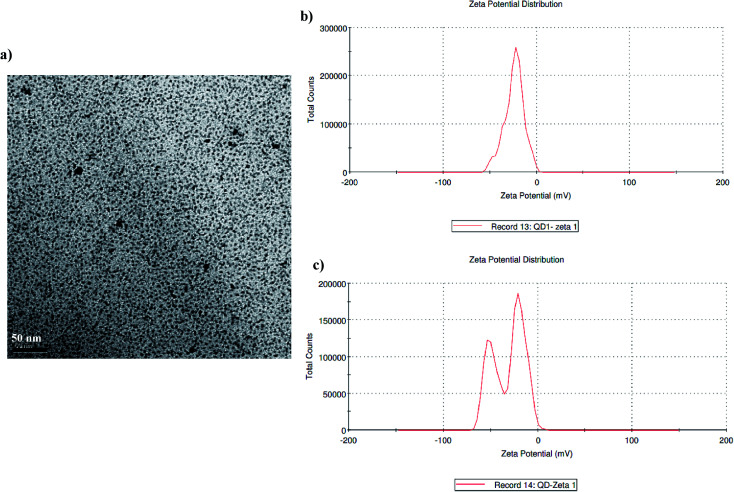
Characterization of the synthesized quantum dots: (a) a high-resolution TEM image of the synthesized Cd/Te quantum dots, (b) DLS data of the synthesized QDs, and (c) DLS data of the surface modified QDs.

Over the past few decades, semiconductor nanocrystals (NCs), also known as quantum dots, have served as attractive biological labels with well documented optical and chemical properties.^26^ In the present study, orange coloured CdTe quantum dots were used as bio-probes for the FRET-based detection of aflatoxin B1 in food samples. The UV-visible absorption spectrum of the prepared QDs depicts a well resolved maximum, illustrating the satisfactory narrow size distribution of the QDs.^27^ The monodispersity of the synthesized QDs was visualized using TEM analysis where uniform QDs with a particle size of ∼4.5 nm were observed (Fig. 4a). The synthesized QDs were characterized using Dynamic Light Scattering (DLS) (Fig. 4b). From Fig. 4c, it can be observed that surface modification of the QDs with TGA does not affect the monodispersity of the solution. The slight variation in particle size between the bare QDs and modified QDs is due to either changes in their surface charges or the attachment of ligands during surface modification.

### 3.3 FRET-based aptasensor and its principle

**Fig. 5:**
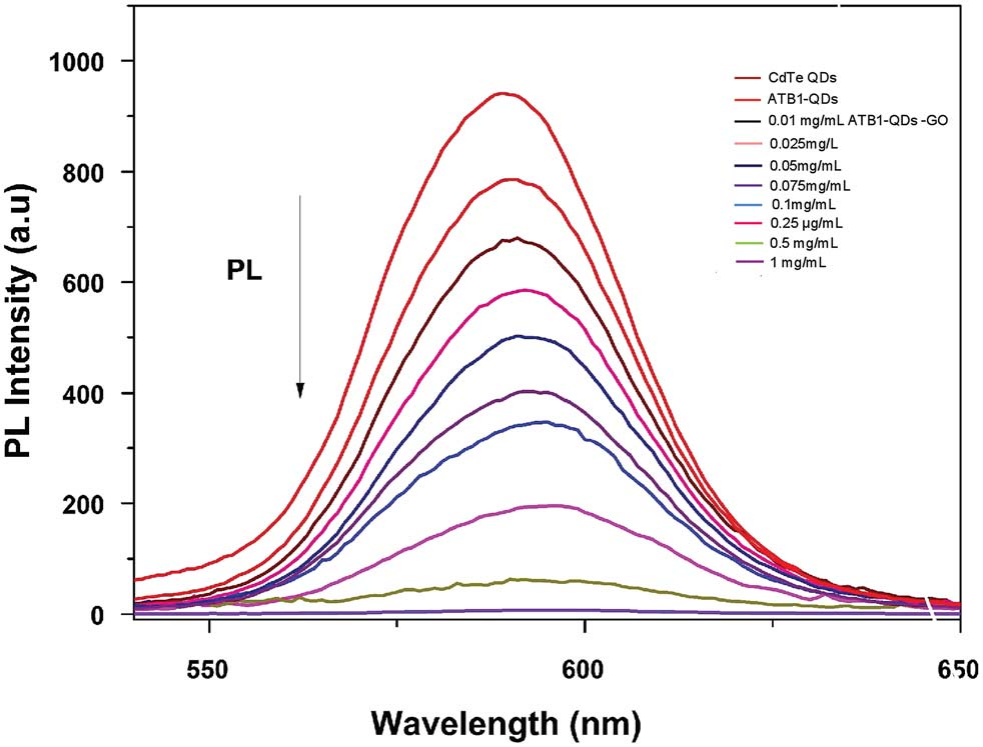
Characterization of graphene oxide–quantum dots: monitoring the fluorescence quenching of quantum dots at various graphene oxide (0.01–1 mg ml^−1^) concentrations using PL spectroscopy.

Unlike usual FRET-based sensors that utilize two different dyes for sensing, quantum dots were employed as labels in this developed FRET aptasensor. In this present study, a graphene oxide based aflatoxin B1 detection platform, based on a turn off/on fluorescence aptasensor, was assembled. The fluorescence of quantum dots can be effectively quenched using graphene due to its excellent quenching of electronically excited states of nanomaterials.^28^ The fluorescence quenching ability of graphene was evaluated using fluorescence spectroscopy; the spectra in Fig. 5 demonstrate the fluorescence quenching of quantum dot labelled aptamers with various concentrations of graphene. The intensity of the fluorescence spectra gradually decreases with increasing graphene concentration. QD conjugated aptamers get adsorbed onto the surface of graphene in the absence of a target, thereby facilitating fluorescence quenching. This highly efficient quenching is achieved through π–π stacking interactions between the bases and GO along with hydrogen bonding interactions between the OH/COOH groups of GO and the OH groups of ssDNA.^29^ These interactions ensure the close proximity of QDs and graphene and efficient quenching. It was also reported by Zhang *et al.* that single-stranded DNA exhibits noncovalent interactions with the aromatic groups present on the surface of nanomaterials. These interactions hold quantum dots near to graphene facilitating high-efficiency energy transfer between graphene and QDs.

Upon addition of aflatoxin B1, fluorescence is gradually recovered as the concentration increases. In the presence of the target, ATB1 aptamers bind to aflatoxin B1 and fluorescence recovery is observed. The recovery of fluorescence is mainly due to the structural variation between single-stranded DNA aptamers and quadruplex DNA. The presence of aflatoxin B1 will induce aptamers adhered to the surface of graphene to form a quadruplex aptamer–aflatoxin B1 complex.^30^ The complex formed possesses a lower binding affinity towards graphene. As a consequence, the fluorophore QDs get far away from the surface of graphene and no energy transfer is facilitated resulting in the recovery of fluorescence. The developed platform is highly sensitive towards aflatoxin B1 and the fact that the target analyte binds to the aptamers rather than the surface of graphene underlies the specificity of the generated aptamers.

### 3.4 Detection of aflatoxin B1 using the FRET-based aptasensor

In comparison to gold nanoparticles used in the study by Sabet *et al.* in 2017, the developed platform is cost-effective and the integrative material used is stable and enhances the sensitivity of the assay towards the target analyte rather than related mycotoxins.

**Fig. 6:**
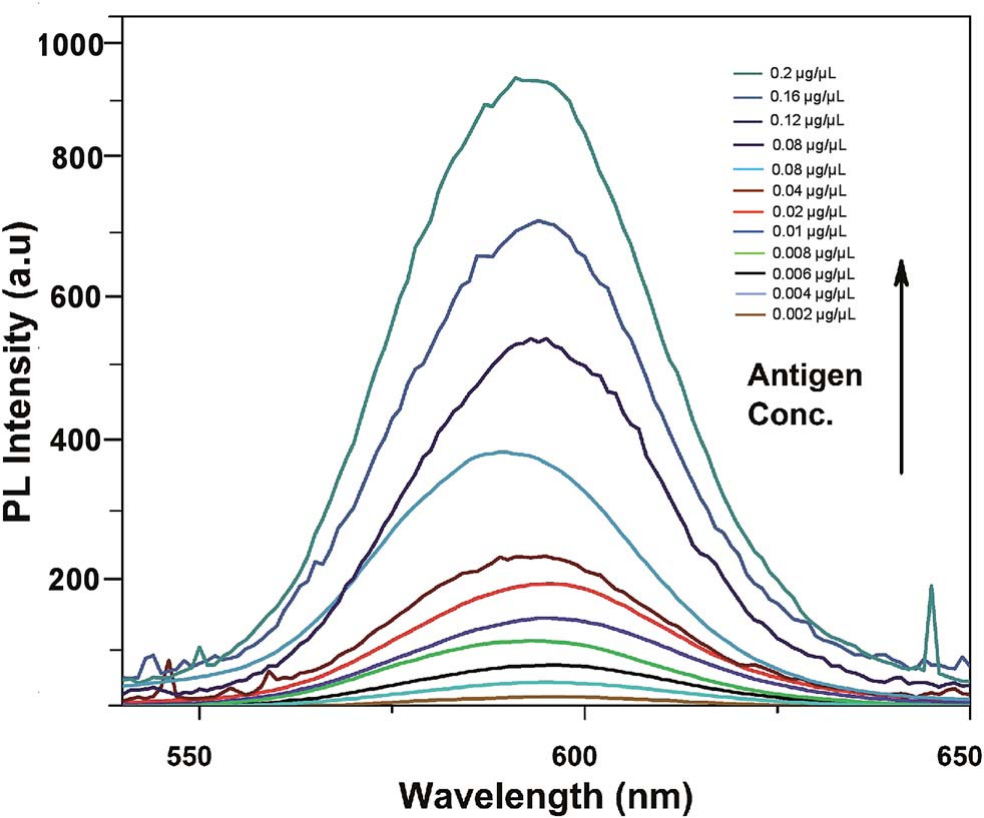
Sensitivity of the developed FRET-based aptasensor platform against various aflatoxin B1 concentrations (0.002 to 0.2 μg μl^−1^).

Fig. 6 shows the fluorescence emission spectra of the aptamer–QD/GO complex after incubation with various concentrations of aflatoxin B1. With the addition of AFB1, fluorescence was markedly resorted starting from a concentration of 0.002 μg μl^−1^, increasing to 0.2 μg μl^−1^. The sensitivity reported in our strategy was quite high compared to those in other chemiluminescence and colourimetric assays. The improved limit of detection might be attributed to the affinity of the generated aptamers towards the target and the improved quenching efficiency of graphene oxide.

### 3.5 Selectivity of the FRET-based aptasensor

**Fig. 7:**
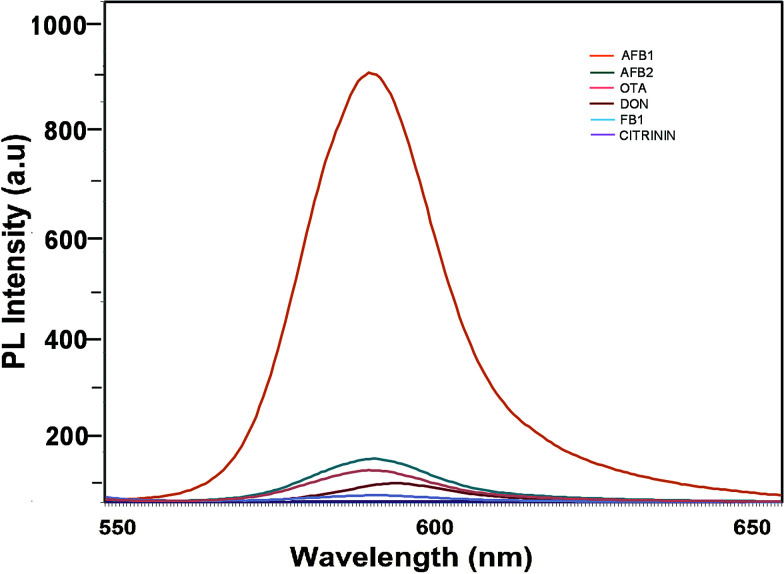
Specificity of the developed FRET-based aptasensor platform against several closely associated mycotoxins.

To determine the sensitivity of the developed assay against the target aflatoxin B1, other mycotoxins, namely AFB B2, G1, AFB G2 and OTA, were evaluated. The fluorescence intensities shown in Fig. 7 highlight the specificity of the developed aptasensor towards aflatoxin B1. Apart from a slight reactivity towards aflatoxin B2, no remarkable changes were observed with other mycotoxin samples. This highlights the specificity of the developed platform against aflatoxin B1 thereby assuring its applicability in field sample analysis.

### 3.6 Detection of aflatoxin B1 in spiked samples using the FRET-based apta-assay

**Fig. 8:**
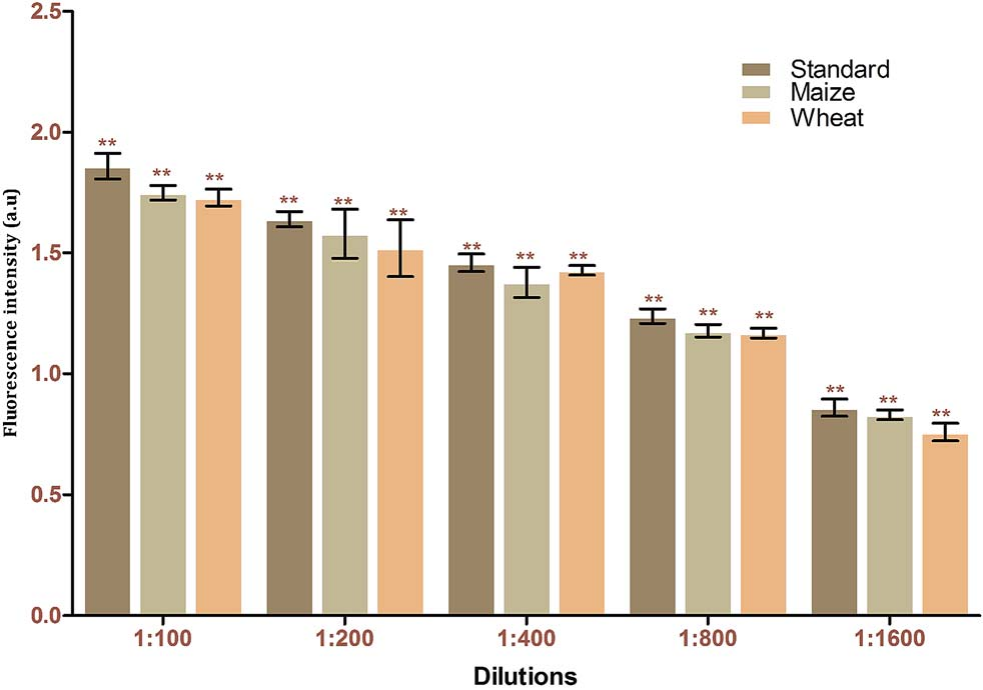
Matrix interference assays of the FRET-based aptasensor against different food matrices (maize and wheat).

To validate the feasibility of the proposed FRET-based aptasensor, wheat and maize samples were spiked with various dilutions of aflatoxin B1. Samples were extracted in duplicate with water–methanol in a ratio of 30 : 70 (v/v). The samples obtained were analysed in triplicate using the developed FRET method. Based on the recovery rate (84–97%), the results obtained from the spiking analysis were in good agreement with the total amount of toxin used for spiking (Table 2). This underlines the applicability of the proposed method for real-time sample analysis. The *p*-value of <0.001 underlines the significance of the recovery percentage directly corresponding to the selectively of the generated aptamers against the target using immuno SELEX. Spiking analysis was carried out in triplicate to eradicate matrix interference effects in the study (Fig. 8). The results obtained were cross verified with results from standard commercially available ELISA kits for AFB1 (Table 3).

**Table 2:** Recovery of AFB1 using aptasensor FRET assay

Spiking level (ng g^−1^)	Mean recovery (ng g^−1^)	Standard deviation of recovery	Mean% recovery
20	19.2	0.53	96.1
10	9.62	0.34	96.2
5	4.3	0.17	87.4
1	0.9	0.02	90
0	NA	NA	NA

**Table 3:** Evaluation of developed aptasensor based FRET against spiked samples

S. no.	Strain name	Aptasensor based FRET	Standard ELISA
1	Maize sample 01	+	+
2	Maize sample 02	+	+
3	Maize sample 03	+	+
4	Maize sample 04	+	+
5	Maize sample 05	+	+
6	Wheat sample 01	+	+
7	Wheat sample 02	+	+
8	Wheat sample 03	+	+
9	Wheat sample 04	+	+
10	Wheat sample 05	+	+

## 4. Conclusion

To date, a reasonable number of studies have been reported in which antibodies are used as bioprobes for mycotoxin detection. However, these bioprobes suffer drawbacks such as high production cost and complicated as well as time-consuming immobilization strategies. The aptamers generated in this study were highly specific towards aflatoxin B1 and do not show significant cross-reactivity against other related mycotoxins. In conjugation with quantum dots and graphene oxide, excellent fluorescence quenching was achieved through the interactions between graphene oxide and the quantum dots, while subsequent fluorescence recovery was achieved in the presence of aflatoxin B1.

In summary, the present study focuses on the development of a highly sensitive and simple FRET-based aptasensor platform for aflatoxin B1 detection. High quenching efficiency was achieved using graphene oxide, thereby enhancing the sensitivity of the developed assay. The developed sensor can detect a wide range of concentrations of the target, from 0.002 μg μl^−1^ (6 nM) to 0.2 μg μl^−1^ (600 μM), with a 4 ng μl^−1^ limit of detection. Spiking analysis discloses the efficiency and robustness of the developed system, with recovery percentages between 83.4 and 97.26%.

## Author contributions

Aswani Kumar (A. K.) and Venkataramana Mudili (V. M.) designed the experiments; A. K., Achuth J. (A. J.) and Renuka R. M. (R. R. M.) performed the experiments; V. M., and Sudhakar P. (S. P.) prepared the manuscript; S. P. and V. M. reviewed the manuscript.

## Conflicts of interest

The authors declare no competing financial interests.

## Supplementary Material
